# Penile metastases of rectal adenocarcinoma after abdominoperineal resection: a case report

**DOI:** 10.1186/s13256-019-2147-z

**Published:** 2019-07-28

**Authors:** Omar Marghich, Yassine Dkhissi, Mohammed Alila, Hicham El Bouhaddouti

**Affiliations:** Department of Visceral Surgery, University Hospital, Hassan 2, Fes, Morocco

**Keywords:** Penile metastasis, Rectal cancer, Corpus spongiosum, Case report

## Abstract

**Background:**

Penile metastases are very rare and arise most frequently from genitourinary cancers. Penile metastases from rectal adenocarcinoma are less common.

**Case presentation:**

We report the case of a 47-year-old North Afican man with penile metastases from a rectal adenocarcinoma, which was discovered 4 months after abdominoperineal resection. A penile biopsy was carried out and established the metastatic nature. He underwent palliative chemotherapy treatment. He was still alive 4 months after diagnosis of penile metastases.

**Conclusion:**

The prognosis of metastasis to the penis is very poor; the best results have been achieved with surgery but only for lesions where metastasis is limited to the penis.

## Introduction

Despite its rich vascularization and the extensive circulatory communication between the neighboring organs, metastatic involvement of the penis is relatively infrequent [1]. Most of the primary tumors that metastasize to the penis originate from pelvic urogenital organs, and rarely from the rectum and rectosigmoid [1–4]. The first report of secondary penile malignancy from an adenocarcinoma of the rectum was defined by Eberth in 1870 [4].

The prognosis of such metastasis is very poor regardless of the treatment options. Treatment is more often palliative than curative [1].

In this case report, we describe a case of penile metastasis secondary to a rectal adenocarcinoma.

## Case presentation

A 47-year-old North Afican man presented with bleeding per rectum and tenesmus of several months’ duration. A rectal examination revealed a mass close to his anal sphincter. Colonoscopy showed a large, ulcerated and multilobulated mass less than 1 cm beyond the anal verge. Multiple biopsies of the rectal mass were obtained. The specimens were sent for pathological examination and returned with findings of moderately differentiated adenocarcinoma.

A computed tomography (CT) scan of his abdomen revealed multiple lymph nodes of the mesorectum measuring less than 5 mm but otherwise no overt metastatic disease.

He received radiochemotherapy then underwent an abdominoperineal resection; there was no clinical or radiological evidence of distant metastasis at the time of resection.

A histopathological examination revealed a moderately differentiated adenocarcinoma of the rectum without lymph node metastasis; the disease was staged as T3 N0 M0. Our patient underwent adjuvant chemotherapy.

He remained well until 4 months later, when he presented with bowel occlusion and urogenital complaints.

**Fig. 1:**
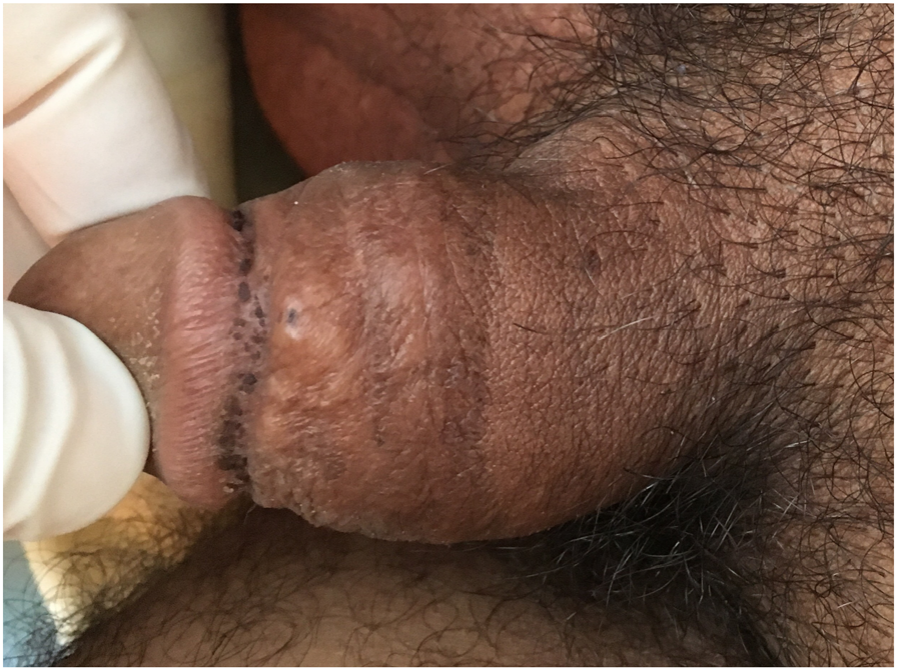
Physical examination of the penis

A physical examination showed a nodule of the corpus cavernosum without visible skin lesions (Fig. 1).

**Fig. 2:**
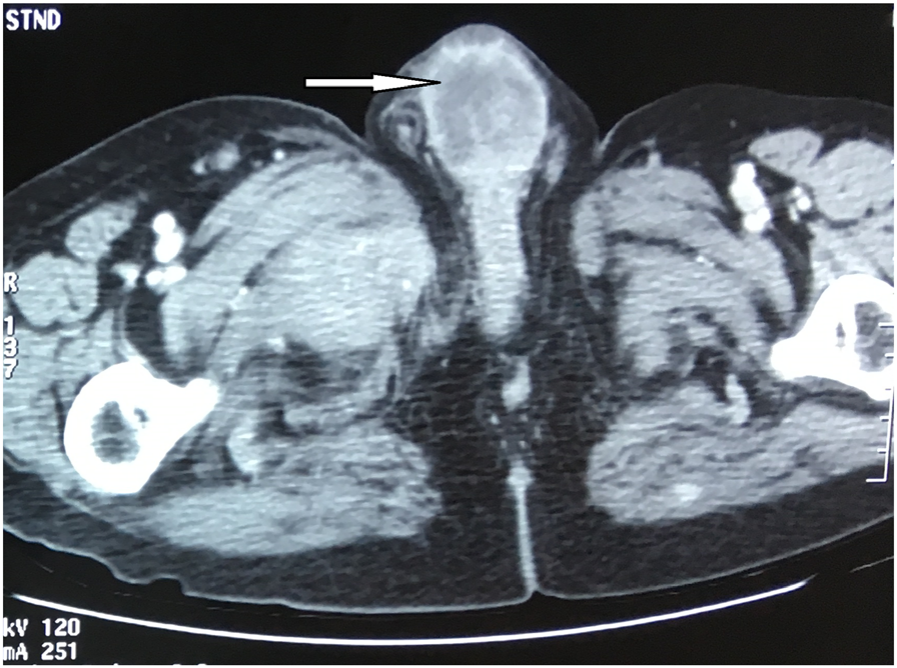
Computed tomography scan showing the penile metastasis (arrow)

A CT scan was performed that showed: lung metastases; external iliac lymph node metastases; lombo-aortic, celio-mesenteric, and inguinal lymph nodes; penile metastases; and bone metastases (Fig. 2).

He received corticotherapy for the bowel occlusion caused by peritoneal carcinomatosis with good evolution then he underwent palliative chemotherapy soon after a biopsy of his penis confirmed the metastatic nature of the lesion. He was still alive 4 months after diagnosis of penile metastases.

## Discussion

Penile metastases are very rare despite the rich vascularization of the penis and its extensive circulatory intercommunications with neighboring organs.

Metastatic involvement of the penis despite its proximity to the rectum mainly originates from the bladder and prostate. Regional lymph nodes, the liver, lungs, and the vertebral column are most likely to be involved in metastasis from rectal cancer [5].

Retrograde venous transportation suggested the main pathways to penis metastasis. The corpus cavernosum is the most common site of penile metastasis. The glans penis and corpus spongiosum are rarely involved [1, 5, 6].

The most common presenting symptoms in order are perineal pain, induration, urethral obstruction, priapism, and hematuria. Metastases can present as well as plaques, wart-like nodules, ulceration, erythema, or induration of the penis [7, 8].

Penile metastasis from rectal adenocarcinoma usually occurs within 2 years after diagnosis of the primary tumor and is frequently associated with dissemination to multiple organs [5, 7–9], for our patient it was 4 months.

Penile metastases are unlikely to be solitary, and treatment should focus on palliative control, which can be achieved by radiation therapy, systemic chemotherapy, or, in selected refractory cases, surgery [5, 7].

However, regardless of the treatment option chosen, prognosis in the setting of penile metastases remains poor. Reported survival usually varies from 7 months to 2 years but some long-term survivals have been seen after aggressive surgical treatment (penile amputation) with the best results noted in patients where penile metastasis was the only site of recurrence [5, 7–11]. Penile amputation is recommended by some authors although the survival benefit is controversial [12]. Others have suggested that it should be reserved for those cases where metastases are isolated to the penis alone [4].

## Conclusion

This report is an additional new case of primary rectal adenocarcinoma with penile metastasis which is rare and usually arises from genitourinary cancers. Despite the advances in cancer therapy within the last decade, the prognosis is still extremely poor. The best results are achieved with surgery when metastasis is limited to the penis.

## Data Availability

All data generated or analyzed during this study are included in this published article.
